# Glutathione Restores the Mechanism of Synaptic Plasticity in Aged Mice to That of the Adult

**DOI:** 10.1371/journal.pone.0020676

**Published:** 2011-05-31

**Authors:** Julie M. Robillard, Grant R. Gordon, Hyun B. Choi, Brian R. Christie, Brian A. MacVicar

**Affiliations:** 1 Department of Psychiatry, Brain Research Centre, University of British Columbia, Vancouver, Canada; 2 Island Medical Program, University of Victoria, Victoria, Canada; Tokyo Medical and Dental University, Japan

## Abstract

Glutathione (GSH), the major endogenous antioxidant produced by cells, can modulate the activity of N-methyl-D-aspartate receptors (NMDARs) through its reducing functions. During aging, an increase in oxidative stress leads to decreased levels of GSH in the brain. Concurrently, aging is characterized by calcium dysregulation, thought to underlie impairments in hippocampal NMDAR-dependent long-term potentiation (LTP), a form of synaptic plasticity thought to represent a cellular model for memory.

Here we show that orally supplementing aged mice with N-acetylcysteine, a precursor for the formation of glutathione, reverses the L-type calcium channel-dependent LTP seen in aged animals to NMDAR-dependent LTP. In addition, introducing glutathione in the intrapipette solution during whole-cell recordings restores LTP obtained in whole-cell conditions in the aged hippocampus. We conclude that aging leads to a reduced redox potential in hippocampal neurons, triggering impairments in LTP.

## Introduction

The tripeptide glutathione (GSH) is the major endogenous antioxidant produced by cells and is critical for the maintenance of the redox potential in the brain. In turn, a healthy redox balance is important to regulate the activity of redox sensitive proteins, such as the N-methyl-D-apartate glutamate receptors (NMDARs) [1]. The NMDAR is the predominant glutamate receptor involved in hippocampal synaptic plasticity and has been shown to be critical for long-term potentiation (LTP) [2] as well as for memory and learning [3]. The activity of the NMDAR can be modulated by the redox environment. Reducing agents have been shown to increase the activity of the NMDARs, while oxidizing agents decrease its activity [1]. During aging, the concentrations of GSH, a potent reducing agent, decrease in the brain [4], a manifestation of the increase in oxidative stress that accompanies normal aging [5]. This increase is thought to be caused by an imbalance between the production of reactive oxygen species (ROS) and the cellular mechanisms responsible for the scavenging of ROS. The age-related increase in oxidative stress likely mediates at least in part the concurrent calcium dysregulation [6]–[8]. In young neurons, the main sources of calcium are influx through NMDARs and voltage-dependent calcium channels such as the L-type calcium channels, as well as release of calcium from intracellular stores. Aging has been associated with a decrease in NMDAR activation as well as an increase in the number of L-type calcium channels [6]. In turn, age-related changes in the contributions of various sources to intracellular calcium levels leads to impairments in calcium-dependent signaling processes such as synaptic plasticity (for a review, see [8]). NMDARs are the main sources of calcium influx for LTP induction, and NMDAR-dependent LTP induction is impaired when weaker stimulation protocols are used in aged animals [9].

These lines of evidence suggest that adding GSH to the diet may represent an attractive candidate for mediating age-related changes in synaptic plasticity. N-acetylcysteine (NAC), a precursor of GSH, can protect the brain against GSH depletion [10]. Here we investigated the possibility that orally supplementing aged mice with NAC could reverse age-induced deficits in NMDAR-dependent hippocampal LTP.

## Results

**Figure 1:**
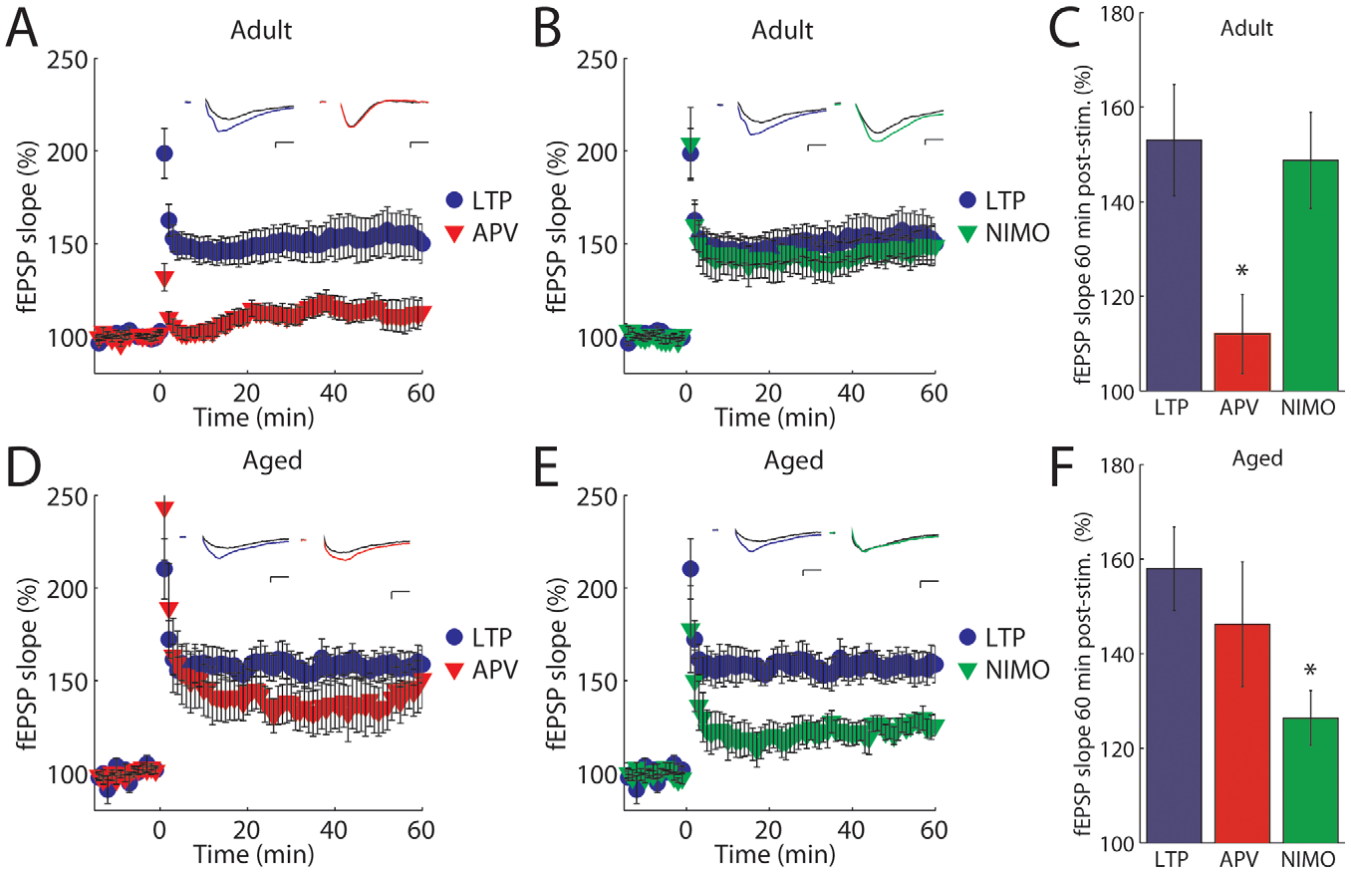
LTP is NMDAR-dependent in adult mice but L-type calcium channel-dependent in aged mice. (**A and B**) CA1 fEPSP slope in adult mice in control conditions (blue •, n = 14) and with APV (50 µM) (red ▾, n = 11) or nimodipine (10 µM) (green ▾, n = 8) in response to HFS applied at t = 0. Inset, averaged fEPSP traces. (**C**) Summary data:in adult, LTP is blocked by APV (*P* = 0.01) but not by nimodipine (*P* = 0.81). (**D and E**) fEPSP slope in aged mice in control (blue •, n = 7) and with APV (red ▾, n = 8) or with nimodipine (green ▾, n = 6) in response to HFS. Inset, averaged fEPSP traces. (**F**) Summary data: in contrast to adult, LTP in aged mice is blocked by nimodipine (*P* = 0.01) but not by APV (*P* = 0.48). Scale bars: 10 ms, 0.4 mV. All data are expressed as mean ± s.e.m.

We initially established the mechanisms underlying LTP in both adult and aged mice. Field excitatory postsynaptic potentials (fEPSPs) were recorded in area CA1 of hippocampal slices from adult (2–4 months old) and aged (14–18 months old) mice. A high-frequency stimulation (HFS) protocol was used to elicit LTP. In the adult hippocampus LTP was blocked by the NMDAR antagonist (2R-amino-5-phosphonovaleric acid (APV) (50 µM) (control: 153±12%, APV: 112±8%; **Figure 1, A and C**) but not by the L-type calcium channel antagonist nimodipine (NIMO) (10 µM) (control: 153±12%, nimodipine: 149±10%; **Figure 1, B and C**). In contrast, LTP in aged mice was not significantly blocked by APV (control: 158±9%, APV: 146±13%; **Figure 1, D and F**) but was significantly decreased by nimodipine (control: 158±9%, nimodipine: 126±6%; **Figure 1, E and F**). Therefore, these findings confirm in a naturally aged mouse model that LTP in adults is NMDAR-dependent whereas LTP in aged mice is L-type calcium channel-dependent.

Consistent with a role for GSH in modifying NMDAR function, brain levels of GSH were significantly lower in the aged mice versus the adult mice (39.8% decrease, n = 5 in each group, *P* = 0.009), demonstrating a positive correlation between GSH and NMDAR-mediated plasticity. To assess the impact of GSH levels on synaptic plasticity, we increased brain GSH levels in aged mice by oral supplementation with NAC (200 mg/kg daily for 21 days; GSH increased by 26.2% compared to aged matched control-fed mice, n = 5 in each group, *P* = 0.041). Imaging of a GSH sensitive dye, monochlorobimane (MCB) in hippocampal cells also indicated that neuronal levels of GSH were increased with oral NAC supplementation (**Figure S1**).

**Figure 2:**
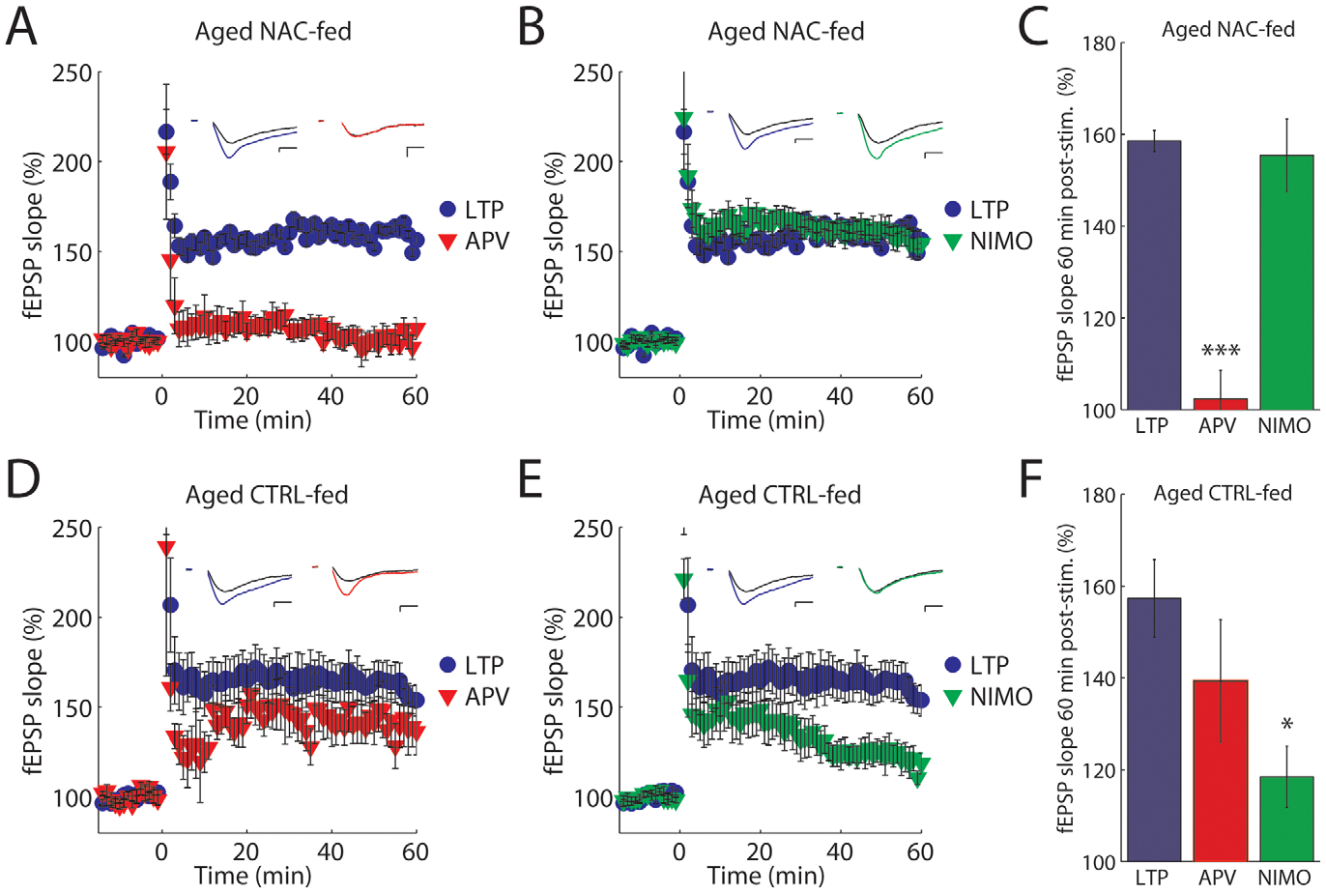
LTP is L-type calcium channel-dependent in aged control-fed mice but NMDAR-dependent in aged NAC-fed mice. (**A and B**) fEPSP slope in aged NAC-fed mice in control (blue •, n = 7) and with APV (red ▾, n = 6) or with nimodipine(green ▾, n = 9) in response to HFS. Averaged fEPSP traces inset. (**C**) Summary data: in NAC–fed mice LTP was blocked by APV (*P*<0.001) but not by nimodipine (*P* = 0.75). (**D and E**) fEPSP slope in aged control-fed mice in control conditions (blue •, n = 8) and with APV (red ▾, n = 5) or with nimodipine (green ▾, n = 7) in response to HFS. Averaged fEPSP traces inset. (**F**) Summary data: LTP in aged, control-fed mice was blocked by nimodipine (**P** = 0.002) but not by APV (*P* = 0.24). Scale bars: 10 ms, 0.4 mV. All data are expressed as mean ± s.e.m.

Redox potential impacts both L-type calcium channels and NMDARs [11], [12]. The activity of NMDARs in particular have been shown to be modulated by the redox state in the environment [12], [13]. This modulation raises the interesting possibility that changing brain GSH levels by supplying a precursor in the diet can modulate synaptic plasticity in the hippocampus. Therefore we repeated our LTP experiments in both NAC-fed aged mice and control-fed aged mice to assess the mechanisms of LTP in mice with altered GSH levels. Remarkably, in the NAC-fed aged mice, LTP was restored back to NMDAR-dependence observed in adult mice, as LTP was completely blocked by APV (control: 159±2%, APV: 102±6%; **Figure 2** **, A and C**) but not by nimodipine (control: 159±2%, nimodipine: 155±8%; **Figure 2** **, B and C**). In contrast, control-fed aged mice retained their L-type calcium channel-dependence because LTP in these mice was not significantly blocked by APV (control: 157±8%, APV: 139±13%; **Figure 2** **, D and F**) but was decreased by nimodipine (control: 157±8%, nimodipine: 118±7%; **Figure 2** **, E and F**). These results demonstrate that LTP in aged control-fed mice is mediated by the same mechanisms observed in aged mice and that oral NAC supplementation in aged mice increases brain GSH levels and restores the NMDAR dependency of LTP seen in adult animals.

**Figure 3:**
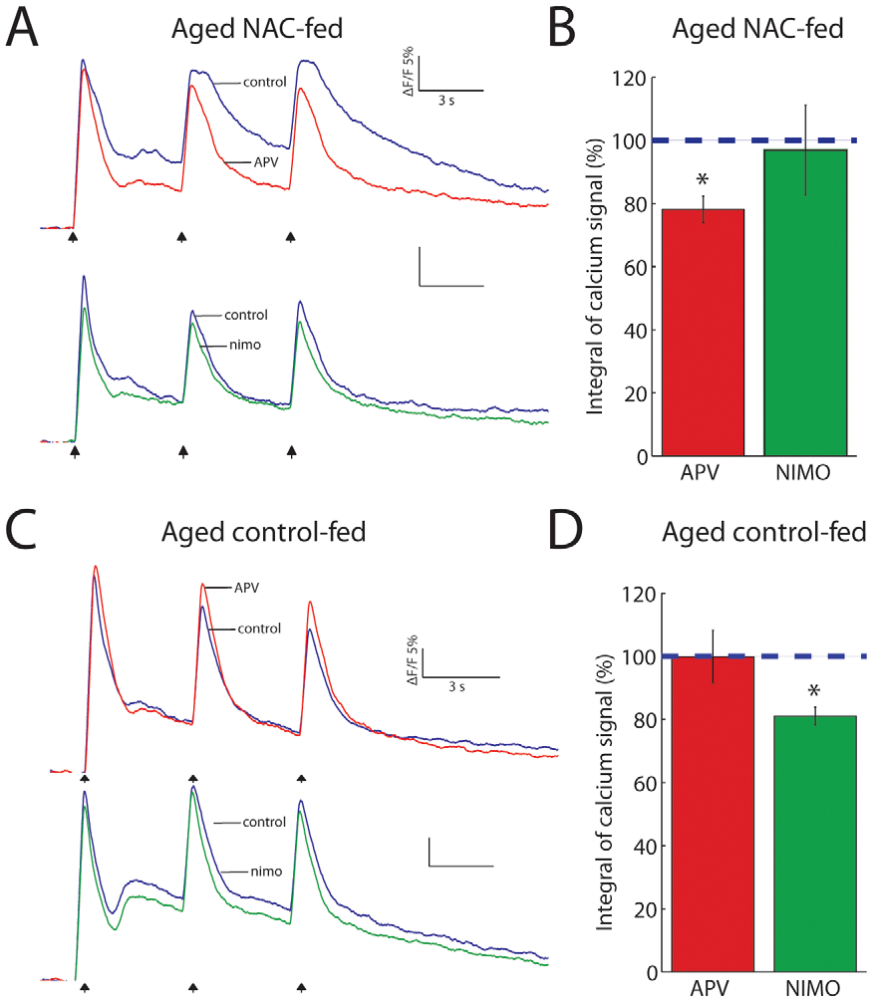
GSH improves NMDAR calcium signals in aged mice. (**A**) Representative calcium signal traces in control (blue) and during APV (red) or nimodipine (green) from an aged NAC-fed mouse in response to HFS. (**B**) Summary data of calcium signal integral (area under the curve). Only NMDAR blockade reduces the calcium signal (APV: n = 6, nimodipine: n = 5, *P* = 0.02). (**C**) Representative calcium signal traces (same color scheme as ***a***) from an aged control-fed mouse in response to HFS. (**D**) Summary data of calcium signal integral (area under the curve). Only nimodipine blockade reduces the calcium signal (APV: n = 5, nimodipine: n = 6, *P* = 0.02).

To further test the impact of increasing brain GSH levels on NMDAR function in aged mice, we used two-photon imaging to determine the contribution of NMDARs to hippocampal dendritic calcium signals elicited by bursts of HFS. Consistent with the mechanisms observed in fEPSPs, we found that calcium signals elicited by HFS were depressed by APV only in NAC-fed aged mice (**Figure 3, A and B**) but not in control-fed aged mice (**Figure 3, C and D**). These results are consistent with the idea that supplementation with a GSH precursor can restore the NMDAR-dependence of hippocampal LTP by enhancing NMDA calcium influx.

It is notoriously difficult to observe hippocampal LTP in older animals in a whole-cell recording configuration. One potential explanation for this difficulty may be the dialysis of important mediators of LTP out of neurons during the procedure. Consistent with the recent observation that intracellular GSH supplementation enhances NMDAR currents [14], we confirmed that LTP could only be observed in whole-cell conditions in aged hippocampal neurons when GSH was added to the intrapipette solution (**Figure S2, A and B**). To further confirm the role of GSH in hippocampal synaptic plasticity, we repeated our whole-cell LTP experiments using adult mice, again in control conditions and with GSH added to the intrapipette solution. We find that similar to whole-cell LTP in aged animals, whole-cell LTP in adult animals could only be observed with GSH in the intrapipette solution (**Figure S2C**).

## Discussion

The data presented here demonstrate that feeding aged mice with the GSH precursor NAC partially restores brain GSH levels and reverses the mechanisms underlying hippocampal LTP in area CA1 from L-type calcium channel dependence (as seen in aged unfed mice) to NMDAR dependence (as seen in adult mice). This switch is associated with improved NMDAR-mediated calcium signals in aged mice in response to a HFS pattern used to evoke LTP. Our findings reproduce and confirm published results by Shankar and colleagues [9]. As well, our findings are supported by evidence for hypofunction of NMDAR in conditions of GSH deficit [15]. Our results are also consistent with the age-related increase in oxidative damage as well as with the evidence for impaired synaptic plasticity in animals with a low GSH content [16].

Over the recent years, the involvement of ROS in hippocampal synaptic plasticity in young and adult animals has been demonstrated. There is evidence for a role for both superoxide [17]–[19] and hydrogen peroxide [20]–[22] in mediating LTP in young animals. In aged animals, there is an increase in the levels of ROS in the brain, and this is suggested to lead to the age-related impairments in hippocampal synaptic plasticity. A number of key LTP components may be modulated by an increase in ROS during aging, ranging from the activity of phosphatases such as calcineurin [23] to a direct modulation of the activity of NMDARs [13]. When assessing the magnitude of LTP in aged mice and comparing with that of adult mice, we did not observe a decrease in the probability of LTP being expressed in our aged mice. While previous studies have shown age-associated deficits in hippocampal LTP, the use of a strong stimulation protocol leads to the activation of L-type calcium channels, which in turn compensate for the deficits in NMDAR-dependent LTP [9].

While the role of NAC as a cysteine donor has been well established [24], NAC can also act as an antioxidant itself, and may modulate the redox properties of NMDARs [25]. However, in our feeding model, oral NAC supplementation leads to a significant increase in hippocampal GSH levels, thus we conclude that NAC is impacting synaptic plasticity at least in part via its cysteine donor properties. Although we cannot unequivocally rule out the contribution of other NAC-mediated mechanisms of antioxidant action, there is no NAC present in the aCSF bathing the slices so a direct effect of NAC is unlikely.

GSH can impact the activity of NMDARs through the modulation of redox-sensitive sites on the extracellular portion of NR1 and NR2A subunits [15]. However, several lines of evidence also suggest that GSH can impact the activity of NMDARs via non-redox mechanisms, namely by acting directly at the NMDAR as an agonist or an antagonist [26]. GSH can displace the binding of both radiolabelled agonists and radiolabelled antagonists of the receptor in synaptic membrane preparations [26], [27]. While it is unclear if GSH acts specifically as an agonist or an antagonist, pharmacological studies report that GSH is more potent at displacing agonists, and therefore, likely acts as an agonist at the NMDAR [26]. However, whether GSH acts as an agonist or an antagonist at the NMDAR may depend on the subunit composition of the receptor or on the concentration of GSH [28]. Given these lines of evidence, it is possible that in our model, GSH increases the activity of NMDARs by acting as an agonist. This mechanism could explain in part our results obtained with whole-cell patch clamp recordings. However, since it has been extensively demonstrated that oxidative stress is increased in models of aging [5] and that NMDARs are more oxidized in this environment [29], we propose that the increase in function of the NMDAR we observed is at least partially due to a GSH-mediated modulation of redox-sensitive sites on the receptors. Further experiments assessing the redox state of the NMDAR during GSH supplementation will be required to determine to what extent this modulation accounts for changes in NMDAR activity in our model.

Whether GSH impacts synaptic plasticity through its reducing properties in the extracellular or intracellular compartment is still unclear. The function of NMDARs can be modulated through the reduction of extracellular cysteine residues [13], arguing for a role for an extracellular action of GSH. However, even though NMDARs do not appear to contain intracellular redox-sensitive sites [30], changes in the intracellular redox environment can alter several enzymatic mechanisms involved in modulating the activity of NMDARs [31]. However, recent work by Bodhinathan et al. (2010) demonstrates that redox mechanisms impacting NMDARs involve the calcium/calmodulin-dependent protein kinase II (CaMKII) and not the major phosphatases or protein kinase C (PKC) [14]. This same work confirms that GSH-mediated reduction of the intracellular environment can potentiate NMDAR currents in aged animals [14]. In addition, GSH has been shown to target the glutamatergic system through the activation of the cystine-glutamate exchanger [32].

While much of our study focused on age-related hypofunction of NMDARs, other types of age-related processes occur in parallel and may impact synaptic function during aging. Like NMDARs, ryanodine receptors (RyRs) can be modulated through the redox state of critical cysteine residues [33]–[35], and this modulation may in turn play a role in regulating synaptic plasticity. A decrease in calcium release from intracellular stores has been shown to increase NMDAR-dependent synaptic plasticity by reducing the slow afterhyperpolarization [36], [37] and increasing NMDA responses [37]. Thus, these results combined with our study supports the hypothesis that changes in the intracellular redox state during aging contributes to calcium dysregulation.

Our results indicate an important role for GSH in maintaining a healthy redox balance in aging neurons and consequently maintaining the NMDAR-dependent LTP. Manipulating GSH levels in the aging brain may be a therapeutic avenue for treating plasticity and memory impairments in age-related diseases.

## Materials and Methods

### Animals and feeding protocol

Adult (2–4 months old) and aged (14–18 months old) male C57BL/6 mice were used for this study. Aged NAC-fed mice were fed once daily for 21 days with NAC at 200 mg/kg incorporated into 1 mL flavored gelatin. Feeding was monitored daily and only the mice that ate the entire portion everyday for the duration of the protocol were included. Aged control-fed mice were fed once daily for 21 days with 1 mL of the same flavored gelatin without NAC. Animals undergoing a feeding protocol were sacrificed and used for experiments the day following their last feeding.

### Hippocampal brain slice preparation

Male C57BL/6 mice were anaesthetized with halothane (Sigma) and decapitated. Hippocampal transverse slices (400 µm) were prepared (Leica vibratome) in a sucrose buffer that contained (in mM) sucrose (230), NaHCO_3_ (26), KCl (2.5), glucose (10), NaH_2_PO_4_ (1.25), MgSO_4_ (10) and CaCl_2_ (0.5) that was constantly oxygenated with 95%O_2_/5%CO_2_. After collection, slices were transferred to an oxygenated (with 95%O_2_/5%CO_2_) artificial cerebrospinal fluid (aCSF) solution for at least 1 hr. The aCSF contained (in mM): NaCl (120), NaHCO_3_ (26), KCl (3), glucose (10), NaH_2_PO_4_ (1.25), MgSO_4_ (1.3) and CaCl_2_ (2).

### Electrophysiology

For extracellular LTP recordings, slices were submerged in a recording chamber perfused with oxygenated aCSF. Field excitatory post-synaptic potentials (fEPSPs) were evoked by stimulation of the Schaffer collateral pathway using a bipolar stimulating electrode. Glass micropipettes filled with aCSF were used to measure CA1 fEPSPs in stratum radiatum. Individual fEPSPs were evoked and recorded every 15 s and a stable 15 min baseline was required in all experiments. After baseline recording, a 100 Hz stimulation protocol (4×50 pulses; 30 s interburst interval) was applied to induce LTP. After application of the conditioning protocol, fEPSPs were again recorded for 1 hr. All drugs were bath-applied 15 min prior to conditioning stimuli. The slopes of the initial phase of the fEPSP waveforms were computed and results were processed for statistical analysis using Clampfit (Axon Instruments, Molecular Devices). Data are presented as means (slopes) and s.e.m. N's indicate number of slices, and each experimental group is comprised of a minimum of five different animals. Statistical significance was determined by assessing the means of the values for the last five minutes of the post-stimuli decay period from two experimental groups using Student's t-test (unpaired).

For whole-cell LTP recordings, whole-cell patch clamp recordings were made using electrodes (4–6 MΩ resistance) filled with a pipette solution containing (in mM): Cs-methanesulfonate (100), CsCl_2_ (25), NaCl_2_ (5), Cs-BAPTA (5), TEA (5), HEPES (20) at pH 7.2. For some experiments, 10 mM glutathione was added to the pipette solution (pH and osmolarity were maintained). Whole-cell voltage-clamp recordings were obtained from CA1 pyramidal cells under microscope guidance using IR-DIC. Membrane potential was clamped at −70 mV. A bipolar electrode was placed on Schaffer collaterals, 250 µm away from the soma of the recorded cell. Synaptic responses were evoked with monophasic voltage pulses every 15 s. Following a stable 10 min baseline, LTP was induced through a well-established paired-stimuli protocol (stimulation frequency at 100 Hz and membrane potential at 5 mV) [38]–[41]. After induction, membrane potential was returned to−70 mV and synaptic responses were monitored every 15 sec for 30 min. Data are presented as means (amplitude) and s.e.m. N's indicate number of slices, and each experimental group is comprised of a minimum of four different animals. Statistical significance was determined by assessing the means of the values for the last five minutes of the post-stimuli decay period from two experimental groups using Student's t-test (unpaired).

### GSH assay

GSH was measured using an assay kit (BioVision, Mountain View, CA). This kit is based on an OPA probe (o-phthalaldehyde), which has a very low fluorescence background, and reacts with the reduced form of GSH and generates a strong fluorescent signal. To obtain total GSH measures, samples were treated with a reducing agent (dithiothreitol) to convert oxidized GSH (GSSG) to reduced GSH. In brief, hippocampal brain slices were homogenized and centrifuged, and the supernatant were transferred to a new tube containing perchloric acid to precipitate the proteins. After centrifugation, the supernatant was collected for the analysis of GSH levels. Fluorescence signals were measured (excitation at 340 nm, emission at 420 nm) using a Gemini Fluorescence Microplate Reader System (Molecular Devices Corporation, Union City, CA). Remaining tissue homogenates were used to measure protein levels to normalize the GSH assay. N's indicate number of animals. Statistical significance was determined by assessing the means of the values for two experimental groups using Student's t-test (unpaired).

### Imaging

Monochlorobimane (MCB) was purchased from Fluka and made as a 100 mm stock solution in Me_2_SO and stored at −20°C. Two-photon imaging of GSH-MCB conjugates in brain slices was achieved using a Coherent (Santa Clara, CA) Chameleon Ti:sapphire ultrafast laser tuned to 780 nm directly coupled to a Zeiss LSM510 system equipped with a 40× (1.0 numerical aperture) objective. Images were acquired using the Zeiss LSM software. Fluorescence was collected using a nondescanned photo-multiplier tube (PMT) for the GSH-MCB signal (512–562 nm). Images (512×512 pixels) were acquired using 8-line averaging between 50 and 100 µm deep into the slice in area CA1. GSH *z*-stacks were acquired from either control-fed mice or NAC-fed mice and fluorescence intensity was normalized to a known Lucifer yellow concentration value inside a pipette positioned in the field of view of the imaged region at the same depth. Regions of interest were delimited to restrict the analysis to the CA1 dendritic field or all astrocytes in the field of few (astrocytes delimited individually). N's indicate number of slices, and each experimental group is comprised of a minimum of four different animals. Statistical significance was determined by assessing the means of the values from two experimental groups using Student's t-test (unpaired).

For calcium imaging of neurons, hippocampal slices from aged mice were loaded with the calcium indicator Rhod-2/AM(10 µM) using the cremophor EL AM dye loading technique developed by Yuste [42]. This technique utilizes Cremophor EL as well as Pluronic acid, two detergents that act to facilitate the solubilization of water-insoluble dyes. The slices were loaded in the dark for a maximum of 45 min at 33 degrees Celsius in aCSF. Rhod-2 fluorescence was visualized by excitation at 835 nm. Calcium transients were measured in the dendrites of CA1 pyramidal neurons by performing line scans in the stratum radiatum of hippocampal slices. A baseline was established by giving 3 bursts of 50 stimulations of the Schaffer collaterals at 100 Hz (interburst interval 5 s), mimicking the LTP conditioning protocol used in electrophysiological experiments, in normal ACSF. Subsequently, either nimodipine (10 µM) or APV (50 µM) was applied to the bath and the conditioning protocol was re-applied to evaluate the contribution of NMDARs and L-type calcium channels to the calcium signal. Fluourescence signals from rhod-2 were analyzed offline using the Zeiss LSM510 software. Fluorescence signals were defined as ΔF/F = [F_1_ - F_0_]/(F_0_), where F_1_ and F_0_ are fluorescence in the dendrites taken from the line scan at any given time point and at the beginning of the experiment respectively. N's indicate number of slices, and each experimental group is comprised of a minimum of four different animals. Statistical significance was determined by assessing the means of the values from two experimental groups using Student's t-test (unpaired).

## Supporting Information

Figure S1 NAC supplementation in aged mice leads to increased levels of GSH in hippocampal neurons. (**A and B**) Brain slice from an aged, control-fed mouse (upper panel) and from an aged, NAC-fed mouse (lower panel), loaded with MCB (60 µM) to visualize the GSH level, showing CA1 region of the hippocampus. (**C and D**) Fluorescence intensity (normalized to a fluorescent standard, see methods) of GSH labeling with MCB in the stratum radiatum (S.R.) (dendritic region, C) and in the astrocyte somas (D). NAC-fed mice showed significantly more GSH in the dendritic region compared with control-fed mice.
(PDF)

Figure S2 (**A**) Whole-cell EPSC amplitude in slices from aged mice recorded with either a normal intrapipette solution (blue •, n = 6) or one supplemented with 10 mM GSH (red ▾, n = 9) in response to HFS stimulation applied at t = 0. (**B**) Summary data. Introducing intracellular GSH restored LTP in aged slices (P = 0.03). (**C**) Introducing intracellular GSH restored LTP in slices from adult mice (P<0.01). All data are expressed as mean ± s.e.m.
(PDF)

## References

[1] Tang LH, Aizenman E (1993). The modulation of N-methyl-D-aspartate receptors by redox and alkylating reagents in rat cortical neurones in vitro.. J Physiol(Lond.).

[2] Bliss TV, Collingridge GL (1993). A synaptic model of memory: long-term potentiation in the hippocampus.. Nature.

[3] Nakazawa K, Quirk MC, Chitwood RA, Watanabe M, Yeckel MF (2002). Requirement for hippocampal CA3 NMDA receptors in associative memory recall.. Science.

[4] Maher P (2005). The effects of stress and aging on glutathione metabolism.. Ageing Res Rev.

[5] Serrano F, Klann E (2004). Reactive oxygen species and synaptic plasticity in the aging hippocampus.. Ageing Res Rev.

[6] Thibault O, Landfield PW (1996). Increase in single L-type calcium channels in hippocampal neurons during aging.. Science.

[7] Burke SN, Barnes CA (2010). Senescent synapses and hippocampal circuit dynamics.. Trends Neurosci.

[8] Foster TC (2007). Calcium homeostasis and modulation of synaptic plasticity in the aged brain.. Aging Cell.

[9] Shankar S, Teyler TJ, Robbins N (1998). Aging differentially alters forms of long-term potentiation in rat hippocampal area CA1.. J Neurophysiol.

[10] Fu A-L, Dong Z-H, Sun M-J (2006). Protective effect of N-acetyl-L-cysteine on amyloid beta-peptide-induced learning and memory deficits in mice.. Brain Res.

[11] Scragg JL, Dallas ML, Wilkinson JA, Varadi G, Peers C (2008). Carbon monoxide inhibits L-type Ca2+ channels via redox modulation of key cysteine residues by mitochondrial reactive oxygen species.. J Biol Chem.

[12] Lipton SA, Choi Y-B, Takahashi H, Zhang D, Li W (2002). Cysteine regulation of protein function--as exemplified by NMDA-receptor modulation.. Trends Neurosci.

[13] Choi YB, Lipton SA (2000). Redox modulation of the NMDA receptor.. Cell Mol Life Sci.

[14] Bodhinathan K, Kumar A, Foster TC (2010). Intracellular redox state alters NMDA receptor response during aging through Ca2+/calmodulin-dependent protein kinase II.. J Neurosci.

[15] Steullet P, Neijt HC, Cuénod M, Do KQ (2006). Synaptic plasticity impairment and hypofunction of NMDA receptors induced by glutathione deficit: relevance to schizophrenia.. Neuroscience.

[16] Almaguer-Melian W, Cruz-Aguado R, Bergado JA (2000). Synaptic plasticity is impaired in rats with a low glutathione content.. Synapse.

[17] Bindokas VP, Jordán J, Lee CC, Miller RJ (1996). Superoxide production in rat hippocampal neurons: selective imaging with hydroethidine.. J Neurosci.

[18] Klann E (1998). Cell-permeable scavengers of superoxide prevent long-term potentiation in hippocampal area CA1.. J Neurophysiol.

[19] Knapp LT, Klann E (2002). Potentiation of hippocampal synaptic transmission by superoxide requires the oxidative activation of protein kinase C.. J Neurosci.

[20] Thiels E, Urban NN, Gonzalez-Burgos GR, Kanterewicz BI, Barrionuevo G (2000). Impairment of Long-term Potentiation and Associative Memory in Mice That Overexpress Extracellular Superoxide Dismutase.. The Journal of Neuroscience.

[21] Auerbach JM, Segal M (1997). Peroxide modulation of slow onset potentiation in rat hippocampus.. J Neurosci.

[22] Kamsler A, Segal M (2003). Hydrogen peroxide modulation of synaptic plasticity.. J Neurosci.

[23] Foster TC (2002). Regulation of synaptic plasticity in memory and memory decline with aging.. Prog Brain Res.

[24] Ercal N, Treeratphan P, Hammond TC, Matthews RH, Grannemann NH (1996). In vivo indices of oxidative stress in lead-exposed C57BL/6 mice are reduced by treatment with meso-2,3-dimercaptosuccinic acid or N-acetylcysteine.. Free Radic Biol Med.

[25] Lavoie S, Murray MM, Deppen P, Knyazeva MG, Berk M (2008). Glutathione precursor, N-acetyl-cysteine, improves mismatch negativity in schizophrenia patients.. Neuropsychopharmacology.

[26] Shaw CA (1998). Glutathione In The Nervous System..

[27] Koller KJ, Coyle JT (1985). The characterization of the specific binding of [3H]-N-acetylaspartylglutamate to rat brain membranes.. J Neurosci.

[28] Janáky R, Ogita K, Pasqualotto BA, Bains JS, Oja SS (1999). Glutathione and signal transduction in the mammalian CNS.. J Neurochem.

[29] Aoyama K, Watabe M, Nakaki T (2008). Regulation of neuronal glutathione synthesis.. J Pharmacol Sci.

[30] Köhr G, Eckardt S, Lüddens H, Monyer H, Seeburg PH (1994). NMDA receptor channels: subunit-specific potentiation by reducing agents.. Neuron.

[31] Sommer D, Coleman S, Swanson SA, Stemmer PM (2002). Differential susceptibilities of serine/threonine phosphatases to oxidative and nitrosative stress.. Arch Biochem Biophys.

[32] Moussawi K, Pacchioni A, Moran M, Olive MF, Gass JT (2009). N-Acetylcysteine reverses cocaine-induced metaplasticity.. Nat Neurosci.

[33] Pessah IN, Kim KH, Feng W (2002). Redox sensing properties of the ryanodine receptor complex.. Front Biosci.

[34] Hidalgo C, Bull R, Behrens MI, Donoso P (2004). Redox regulation of RyR-mediated Ca2+ release in muscle and neurons.. Biol Res.

[35] Bodhinathan K, Kumar A, Foster TC (2010). Redox sensitive calcium stores underlie enhanced after hyperpolarization of aged neurons: role for ryanodine receptor mediated calcium signaling.. J Neurophysiol.

[36] Gant JC, Sama MM, Landfield PW, Thibault O (2006). Early and simultaneous emergence of multiple hippocampal biomarkers of aging is mediated by Ca2+-induced Ca2+ release.. J Neurosci.

[37] Kumar A, Foster TC (2004). Enhanced long-term potentiation during aging is masked by processes involving intracellular calcium stores.. J Neurophysiol.

[38] Hjelmstad GO, Nicoll RA, Malenka RC (1997). Synaptic refractory period provides a measure of probability of release in the hippocampus.. Neuron.

[39] Kato K, Clifford DB, Zorumski CF (1993). Long-term potentiation during whole-cell recording in rat hippocampal slices.. Neuroscience.

[40] Manabe T, Wyllie DJ, Perkel DJ, Nicoll RA (1993). Modulation of synaptic transmission and long-term potentiation: effects on paired pulse facilitation and EPSC variance in the CA1 region of the hippocampus.. J Neurophysiol.

[41] Perkel DJ, Nicoll RA (1993). Evidence for all-or-none regulation of neurotransmitter release: implications for long-term potentiation.. J Physiol (Lond.).

[42] Ikegaya Y, Le Bon-Jego M, Yuste R (2005). Large-scale imaging of cortical network activity with calcium indicators.. Neurosci Res.

