# Supplementary material for: Glutathione Restores the Mechanism of Synaptic Plasticity in Aged Mice to That of the Adult
Source: PLoS One. 2011 May 31;6(5):e20676. doi: 10.1371/journal.pone.0020676 (PMC3105108; doi:10.1371/journal.pone.0020676)
Supplement: Figure S1 — NAC supplementation in aged mice leads to increased levels of GSH in hippocampal neurons. (A and B) Brain slice from an aged, control-fed mouse (upper panel) and from an aged, NAC-fed mouse (lower panel), loaded with MCB (60 µM) to visualize the GSH level, showing CA1 region of the hippocampus. (C and D) Fluorescence intensity (normalized to a fluorescent standard, see methods) of GSH labeling with MCB in the stratum radiatum (S.R.) (dendritic region, C) and in the astrocyte somas (D). NAC-fed mice showed significantly more GSH in the dendritic region compared with control-fed mice. (PDF) [file pone.0020676.s001.pdf]

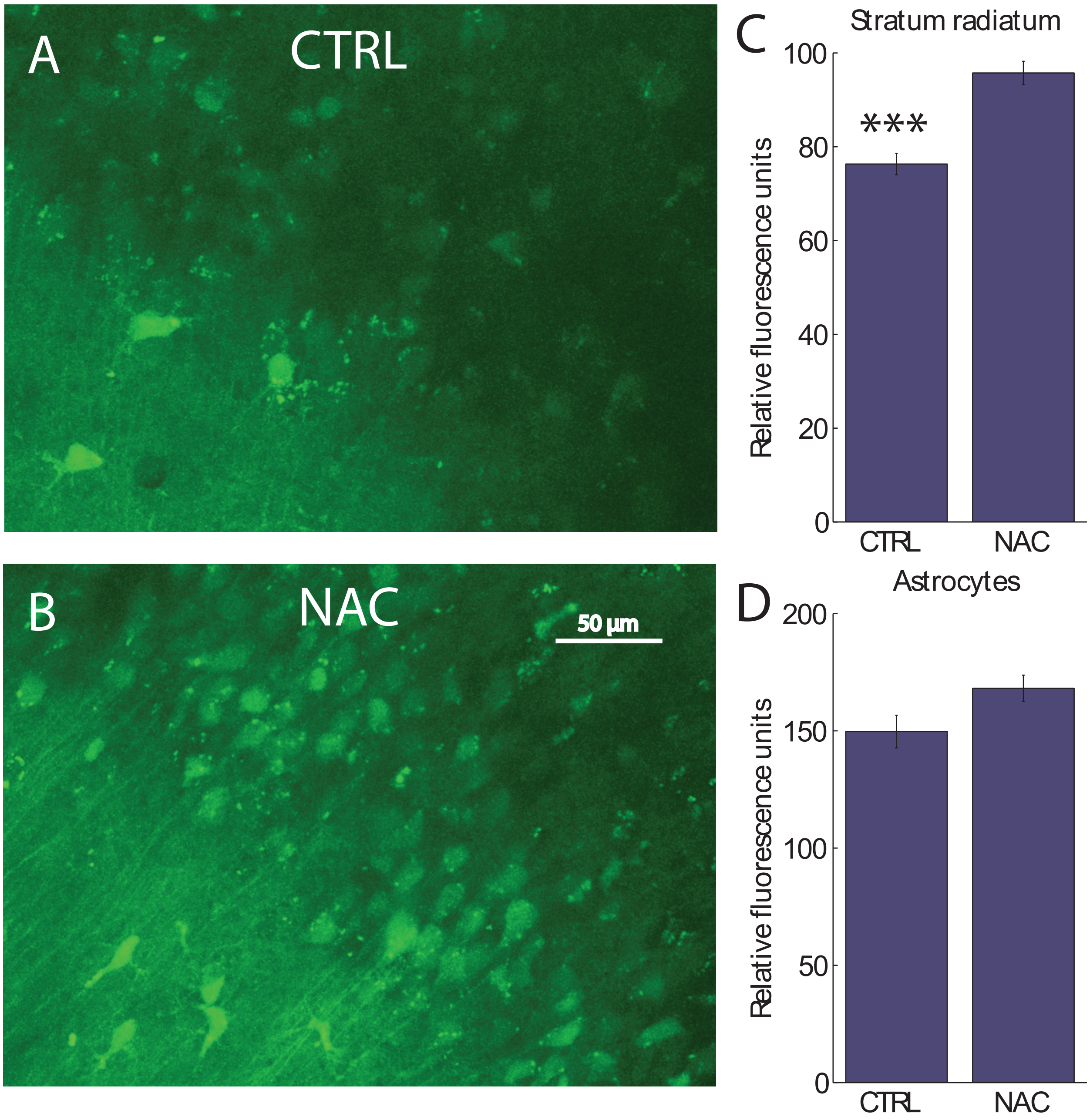

Supplementary Figure 1. NAC supplementation in aged mice leads to increased levels of GSH in hippocampal neurons. **A and B** Brain slice from an aged, control-fed mouse (upper panel) and from an aged, NAC-fed mouse (lower panel), loaded with MCB (60 µM) to visualize the GSH level, showing CA1 region of the hippocampus. **C and D** Fluorescence intensity (normalized to a fluorescent standard, see methods) of GSH labeling with MCB in the stratum radiatum (S.R.) (dendritic region, **C**) and in the astrocyte somas (**D**). NAC-fed mice showed significantly more GSH in the dendritic region compared with control-fed mice.
