# Supplementary material for: Glutathione Restores the Mechanism of Synaptic Plasticity in Aged Mice to That of the Adult
Source: PLoS One. 2011 May 31;6(5):e20676. doi: 10.1371/journal.pone.0020676 (PMC3105108; doi:10.1371/journal.pone.0020676)
Supplement: Figure S2 — (A) Whole-cell EPSC amplitude in slices from aged mice recorded with either a normal intrapipette solution (blue •, n = 6) or one supplemented with 10 mM GSH (red ▾, n = 9) in response to HFS stimulation applied at t = 0. (B) Summary data. Introducing intracellular GSH restored LTP in aged slices (P = 0.03). (C) Introducing intracellular GSH restored LTP in slices from adult mice (P<0.01). All data are expressed as mean ± s.e.m. (PDF) [file pone.0020676.s002.pdf]

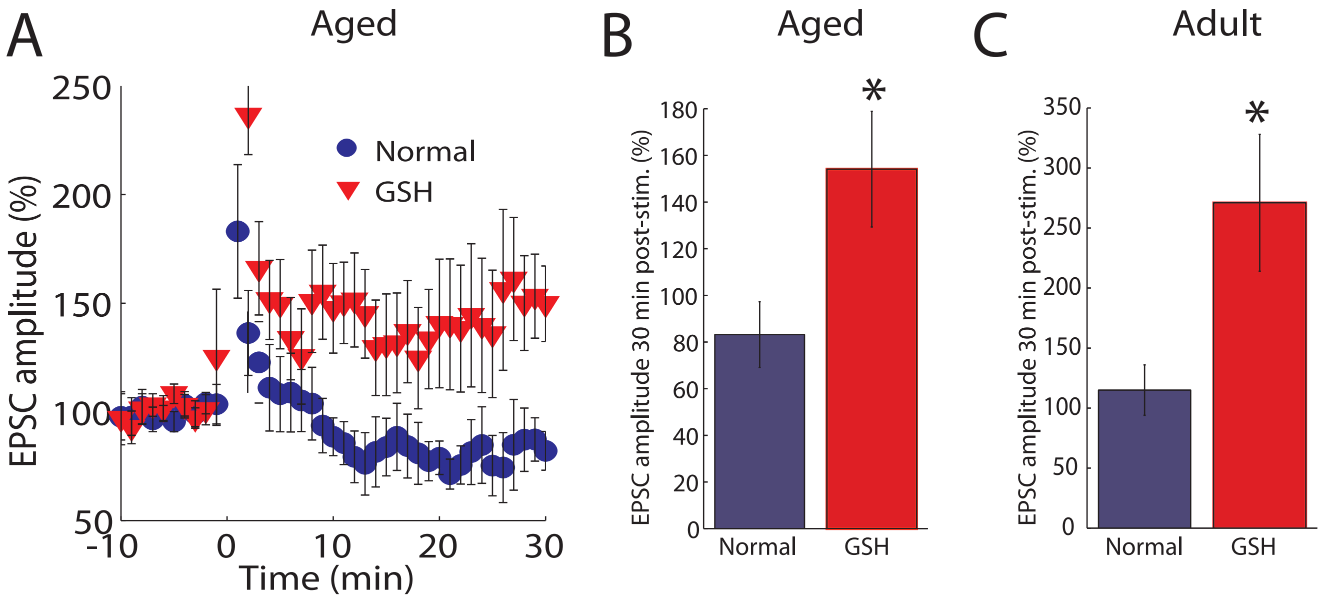

Supplementary Figure 2. (a) Whole-cell EPSC amplitude in slices from aged mice recorded with either a normal intrapipette solution (●, n=6) or one supplemented with 10 mM GSH (▾, n=9) in response to HFS stimulation applied at t=0. (b) Summary data. Introducing intracellular GSH restored LTP in aged slices (P=0.03). (c) Introducing intracellular GSH restored LTP in slices from adult mice (P<0.01). All data are expressed as mean ± s.e.m.
